# The transgenic cloned pig population with integrated and controllable GH expression that has higher feed efficiency and meat production

**DOI:** 10.1038/srep10152

**Published:** 2015-05-11

**Authors:** Huiming Ju, Jiaqing Zhang, Lijing Bai, Yulian Mu, Yutao Du, Wenxian Yang, Yong Li, Anzhi Sheng, Kui Li

**Affiliations:** 1 Key Laboratory of Farm Animal Genetic Resources and Germplasm Innovation of Ministry of Agriculture, Institute of Animal Science, Chinese Academy of Agricultural Sciences, Beijing, 100193, P. R. China; 2 College of Veterinary Medicine, Yangzhou University, Yangzhou 25009, Jiangsu, P. R. China; 3 Department of Animal Genetics, Breeding and Reproduction, College of Animal Science and Technology, Nanjing Agricultural University, Nanjing, 210095, P. R. China; 4 BGI-Shenzhen, Bei Shan Road, Yantian, Shenzhen, 518083, P. R. China

## Abstract

Sustained expression of the GH gene has been shown to have detrimental effects on the health of animals. In the current study, transgenic founder pigs, with controllable pig growth hormone (pGH) expression, were cloned via the handmade cloning method (HMC), and pGH expression levels were examined at the cellular and organismal levels. The serum pGH levels in 3 founder male pigs were found to be significantly higher after induction with intramuscular injection of doxycycline (DOX) compared to baseline. A daily dose of DOX was administered via feed to these animals for a period of 65 to 155 days. The growth rate, feed efficiency and pGH serum concentration increased in the DOX-induced transgenic group compared with the other groups. 8 numbers of animals were euthanized and the dressing percentage, loin muscle and lean meat percentage were significantly higher in the DOX-induced F1 transgenic group compared with the other groups. In this study a large population of transgenic pigs, with integrated controllable expression of a transgene, was obtained. The transgenic pigs were healthy and normal in terms of reproductive capability. At the same time, feed efficiency was improved, production processes were accelerated and meat yield was increased.

The production of transgenic pigs is very important for the promotion of economic development. In agriculture, the use of transgenes can increase the growth rate of animals, improve feed efficiency, improve carcass composition, increase disease resistance and enhance reproductive performance^1^,^2^,^3^. In medicine, transgenic pigs are often used to develop suitable models of various human diseases, to produce pharmaceutical proteins for biomedical research^4^,^5^ and to provide donor organs for human transplantation^6^,^7^,^8^. Thus, transgenic pigs are extremely useful and valuable.

The first cloned sheep, Dolly, was cloned from cultured mammary somatic cells and was born in 1996^9^. This event initiated experimentation in mammalian somatic cell nuclear transfer (SCNT). This technology has been successfully applied to many mammalian species and has resulted in cloned cattle^10^,^11^, mice^12^, goats^13^, pigs^14^,^15^, rabbits^16^, cats^17^ and mules^18^. The success of porcine nuclear transfer technology, combined with transgenic technology, has resulted in the production of transgenic pigs^19^,^20^,^21^. However, the low efficiency of gene transfer and the high cost have limited the use of the SCNT method. Since its development, studies have demonstrated the unique advantages and prospective applications of the handmade cloning (HMC) method^22^,^23^,^24^. Compared with traditional SCNT, HMC costs less, is more efficient, and is easier to use and apply to large animals, including domestic animals^25^,^26^,^27^.

Controllable expression of a foreign gene is a developing area in the field of transgenics. Researchers have built a series of gene expression control systems based on an in-depth understanding of the biological mechanisms of gene regulation. Of these, the Tet-On expression system, first established by Gossen and Bujard in 1992, is now the most widely used^28^. The Tet-On system is used to express inducible genes in cells *in vitro*^29^, as well as in transgenic mice^30^,^31^,^32^, large animal models^33^ and other mammals^34^,^35^. Currently, it is the best regulatory system for inducing gene expression. The system uses two vectors for regulating gene expression. In order to use the Tet-On system, two transfections and two selections are required in order to perform functional gene research at the cellular level and separate populations of rtTA and TRE transgenic animals are needed. These animals are then hybridized to select offspring that concurrently express target genes and regulatory genes. The system requires a lot of work and a long experimental timeframe^33^. To address the above-mentioned problems, many researchers have integrated both a regulatory and a target gene on the same expression vector in order to construct a single vector-induced expression system. These expression systems have been successfully used in cells, mice, and pigs^36^,^37^,^38^,^39^.

Growth hormone (GH) not only promotes animal growth, but improves feed efficiency and lean meat percentage. GH transgenes can markedly enhance the growth rate and feed efficiency of animals, as has been confirmed in both transgenic mice and pigs^2^,^40^,^41^. However, over-expression of exogenous GH may cause pathological changes, such as arthritis, stomach ulcers, heart hypertrophy, liver and kidney disease, infertility, as well as other conditions^2^,^42^,^43^.

Healthy, transgenic pigs, with controllable GH expression have the potential for a wide range of applications. For instance, improvements in the growth rate and feed efficiency of pigs could effectively reduce the pressure of food shortages by increasing the availability of pork.

In the current study, the Tet-On system of integrated GH expression was used for the preparation of transgenic pigs via the simple HMC system. An integrated controllable pGH expression vector was constructed and a transgenic pig population, with integrated controllable expression, was developed using the HMC method. Results of this study will augment research examining the effects of regulating exogenous GH gene expression on such things as animal health, reproductive performance, growth and development. In addition, an analysis of the growth regulation mechanism is presented. Results of this study lay the foundation for the safe and efficient preparation of transgenic, GH-expressing, domestic animals and indicate that controllable expression can be successfully used in transgenic livestock.

## Results

### Induction efficiency of transgenic cells

**Figure 1:**
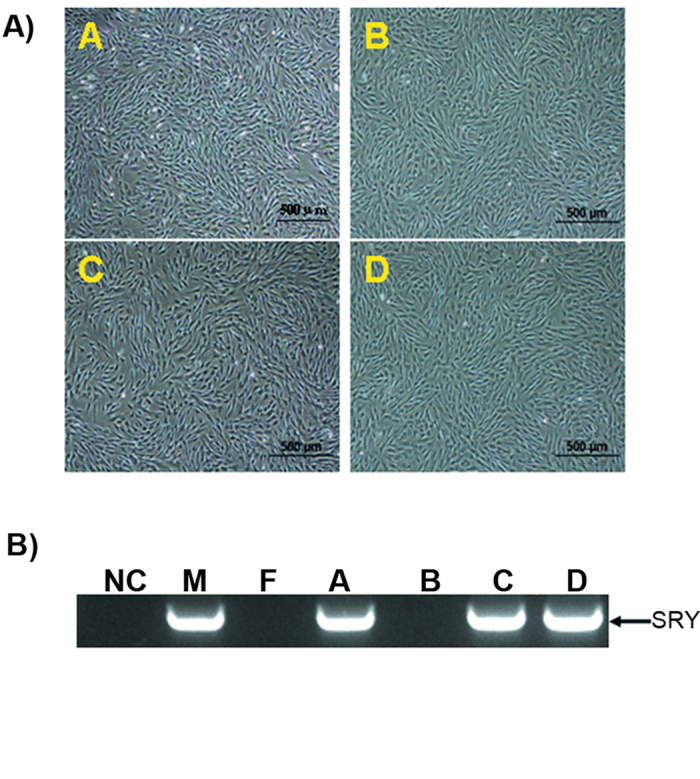
Separation and sex determination of PEF cells. **A**) PEF cells were separated from porcine fetuses. **B**) Detection of the SRY gene from separated cell lines. M stands for DL2000 DNA marker, blank control (NC) using water as the amplification template. The blots have been cropped to focus on the bands of interest. See Supplementary Fig. S1 for full-length gels. M and F are male and female control cells; **A**, **B**, **C** and **D** are cell lines.

**Figure 2:**
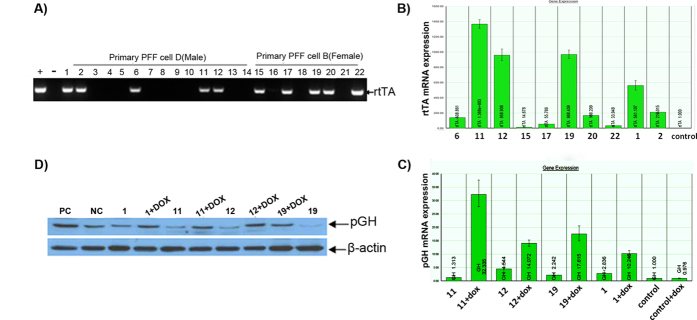
Identification and induction of transgenic donor cell clones. **A**) rtTA gene detection in genomic DNA of G418 positive PEF cell lines. Lane M, DL2000 DNA marker; positive control (+, PC) and negative control (−, NC) using pTTGH plasmid DNA and untransfected PEF cell genomic DNA as templates, respectively. **B**) QRT-PCR analysis of rtTA expression in rtTA integrated cell clones. The cell clones with the highest rtTA mRNA expression levels are circled. **C**) Real-time PCR analysis of pGH mRNA expression in cell clones before and after DOX induction. The pGH mRNA expression levels in PEF cell clones NO.11 and NO.19 have low basic pGH expression levels and higher induction efficiency. **D**) Western blot analysis of pGH protein expression in cell clones before and after DOX induction. The positive control (PC) was the cells transfected with pcDNA-GH plasmid; the negative control was the normal cells. The gradation analysis method was used to compare the pGH induction and expression efficiency in PEF cell lines. The blots have been cropped to focus on the bands of interest. See Supplementary Fig. S2-3 for full-length gels.

Four well-grown porcine embryo fibroblast (PEF) cell lines were established from 4 porcine fetuses (Fig. 1A). PCR was used to detect the genders of the cell lines. The PCR fragment size was 524 bp, as shown by 1% agarose gel electrophoresis (Fig. 1B). Sequencing results indicated that this fragment was the targeted region of the SRY gene. Cell lines A, C, and D were developed from male fetal pigs and B was developed from a female fetal pig. PEF cell lines B and D were chosen for transfection with the foreign gene. After transfection and selection, 14 male cell clones and 8 female cell clones were screened with G418. The cell clones were identified based on PCR amplification of a 482 bp rtTA fragment. Four male and 5 female cell clones were screened for use in the production of transgenic cell lines (Fig. 2A). Real-time PCR analysis of rtTA expression was performed using pGAPDH as a house-keeping gene. Cell clones (NO. 1, 11, 12, 19) with the highest rtTA expression efficiencies were chosen and their induction efficiencies were determined (Fig. 2B). The pGH mRNA expression level increased with DOX induction in all cell clones examined. Cell clones (NO. 11 and NO. 19) demonstrated significantly increased GH expression levels than those of control groups following the DOX induction while the increase disappeared in the absence of DOX induction (Fig. 2C). Results of Western blot analysis verified that the expression of the GH protein was successfully induced in 3 of the selected cell clones (NO. 1, 11, 19) (Fig. 2D). Cell clones NO. 11 and NO. 19 were selected for HMC.

### Transgene copy number and insertion site

**Figure 3:**
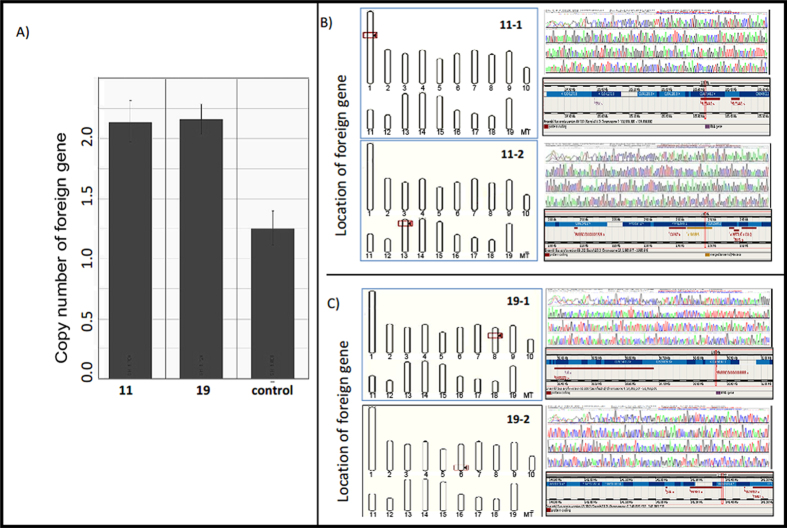
Gene copy numbers and insertion sites in donor cell clones. **A**) Foreign gene copy numbers were measured with a modified QRT-PCR method. Non-transgenic Large White pig genomic DNA was used as the control QRT-PCR template. The transgenic donor cell clones NO.11 and NO.19 were used as QRT-PCR templates. **B**, **C**) The locations of foreign genes in the transgenic donor cells. Specific DNA fragments obtained using the genomic walking method were recovered and sequenced. **B**) The foreign gene in donor cell clone NO.11 was located on chromosomes 1 and 13. **C**) The foreign gene in donor cell clone NO.19 was located on chromosomes 6 and 7.

The copy numbers of transgenic donor cell clones, NO. 11 and NO. 19, were measured using modified QRT-PCR. One non-transgenic, Large White pig was chosen as a control and was found to have 2 copies of endogenous GH. Cell clones NO. 11 and NO. 19 each had 4 copies of the GH gene (Fig. 3A). The specific DNA fragments were gel extracted and sequenced according to the manufacturer’s instructions. Comparison of sequencing results with porcine genomic data from the *ensembl* website ( http://www.ensembl.org/index.html) indicated that 2 copies of the foreign GH gene from transgenic donor cell clone NO. 11 (from a male fetus) were located on chromosomes 1 (Chr1:105336697) and 13 (Chr13:2490397) (Fig. 3B) and 2 copies of the foreign GH gene from transgenic donor cell clone NO. 19 (from a female fetus) were located on chromosomes 8 (Chr8:50866342) and 6 (Chr6:141389908) (Fig. 3C).

### Generation of F0 transgenic pigs

**Figure 4:**
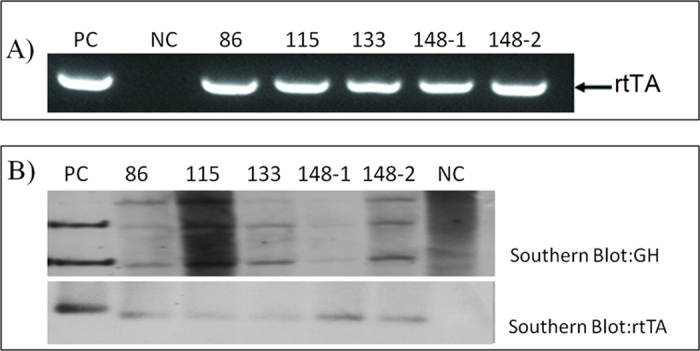
Identification of F0 transgenic cloned pigs. A) Detection of the rtTA gene in genomic DNA from blood samples of the 5 cloned pigs (lanes 4–8) and a non-transgenic pig (lane 2). Lane PC, using a pTTGH DNA fragment as a template; lane NC, using genomic DNA from non-transgenic pigs as a template. B) Transgenic pigs were identified using Southern blot and a Dig-labeled GH probe was used to hybridize the genomic DNA from ear tip tissue of transgenic pigs. After digestion with *Eco*RI, the endogenous GH (pGH1 in B) band and part of the pTTGH fragment (pGH2 in B) were detected in transgenic pigs. rtTA was also detected. The blots have been cropped to focus on the bands of interest. See Supplementary Fig. S4-5 for full-length gels.

The percentages of male and female reconstructed embryos, which developed to the blastocyst stage 6 days after reconstruction, were 45.8% (830/1814) and 42.1% (553/1313), respectively. Nine recipient sows received a total of 122, 82, 111, 131, 119, 92, 92,116 and 83 blastocysts from a mixture of day 5 and 6 blastocysts. Three recipient sows became pregnant and delivered 6 pigs from male donor cells (cell clone NO. 11), including 1 dead fetus, 1 weakened fetus (pig NO. 140), 1 pig that died at delivery due to suffocation and 3 surviving, healthy pigs (pigs NO. 86, 115 and 133). Two recipient sows became pregnant and delivered 6 pigs from female donor cells (cell clone NO. 19), including 4 dead fetuses and 2 surviving, healthy pigs (pigs NO.148-1 and 148-2). The birth weights of the surviving pigs ranged from 620 g to 1250 g and weight gain followed the normal growth curve in all surviving pigs. The cloned pigs were identified by PCR and Southern blot. The PCR results indicated that all of the cloned pigs possessed an amplified 482 bp rtTA PCR product (Fig. 4A). The Southern blot hybridization results were consistent with those of PCR. After digestion with restriction enzyme *Eco*R I, only the transgenic pigs with the pGH gene had detectable amounts of the other GH fragment (Fig. 4B). All cloned pigs were positive for the rtTA probe (Fig. 4B).

### Inducible expression of pGH in cells from F0 transgenic pigs

**Figure 5:**
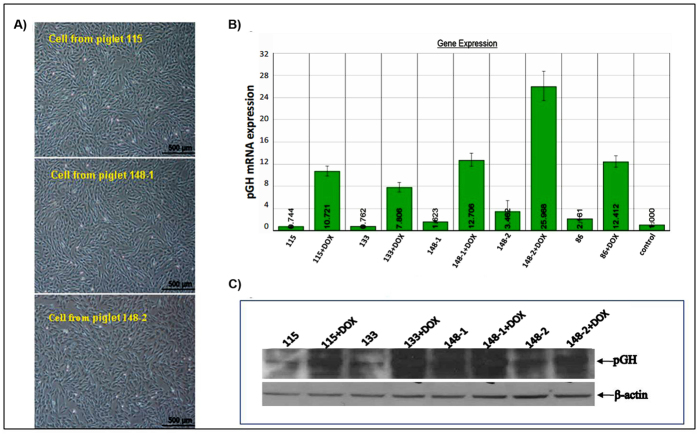
Expression of pGH in cells from F0 transgenic pigs in the presence and absence of DOX. A) Ear-tip fibroblasts were separated from partial transgenic pigs. B) QRT-PCR analysis of pGH mRNA expression in ear-tip fibroblast cells from transgenic pigs. C) Western blot analysis of pGH protein detection in cells from transgenic pigs. After DOX induction, pGH protein levels increased significantly compared with those in the control and the non-induced groups. The blots have been cropped to focus on the bands of interest. See Supplementary Fig. S6 for full-length gels.

The inducible expression of GH was detected in cells from the transgenic pigs in order to preliminarily assess the functional activity and controllability of the exogenous gene. Five different fibroblast populations from 5 founder cloned transgenic pigs were isolated (Fig. 5A). The expression levels of pGH mRNA and protein in cells from the 5 founder pigs had significantly increased after induction compared with the corresponding levels before induction, as indicated by an induction test (Fig. 5B,C).

### Inducible expression of pGH in F0 transgenic pigs

**Figure 6:**
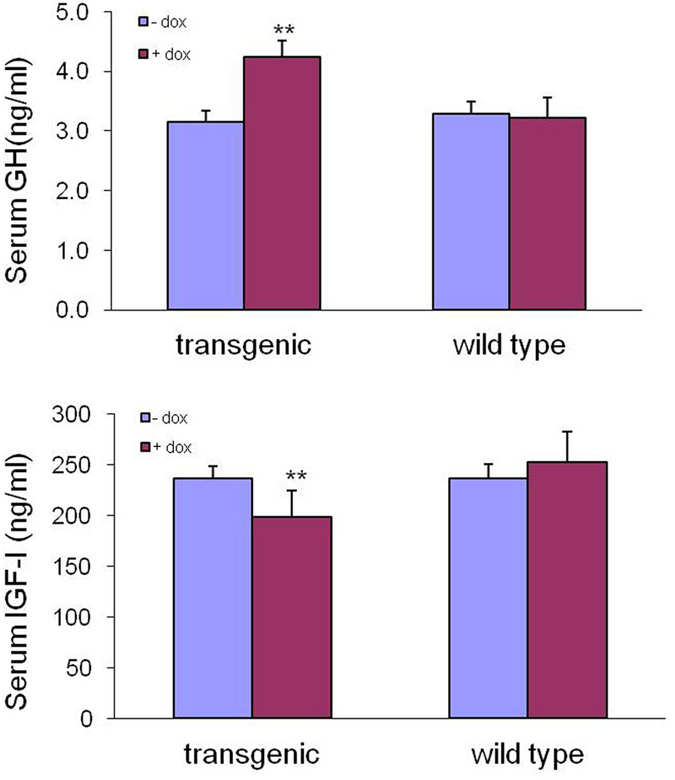
Serum pGH and IGF-1 levels in F0 transgenic pigs before and after DOX induction. **A**) Serum GH detection. Before DOX induction, the average serum pGH concentrations of 3 cloned transgenic pigs (86, 115, and 133) were significantly lower than the average concentrations after DOX induction. **B**) Serum IGF-1 detection. After DOX induction, the average serum IGF-1 concentrations of 3 cloned transgenic pigs decreased significantly compared with the average serum concentrations before DOX induction. The control pigs showed no significant differences in GH and IGF-1 levels, regardless of whether they were induced with DOX.

The pGH levels were detected before and after DOX induction to determine whether the exogenous gene was active. IGF-1 was detected simultaneously to examine the variable effect of pGH on pig growth. Blood samples were collected at 6, 12 and 18 hours, via the anterior vena cava after DOX induction and the pGH values in the serum were measured and averaged at each time point. The pGH levels were significantly higher after DOX induction compared with the pGH levels before induction (3.149 ± 0.197 ng/ml vs. 4.240 ± 0.204 ng/ml; P < 0.05) (Fig. 6A).Serum IGF-1 values declined significantly after DOX induction compared with values before induction (197.964 ± 14.243 ng/ml vs. 236.658 ± 11.287 ng/ml; P < 0.01) (Fig. 6B). There was no significant difference in the serum levels of pGH or IGF-1 before and after DOX induction in wild type pigs (3.283 ± 0.272 ng/ml vs. 3.223 ± 0.345 ng/ml and 236.444 ± 26.297 ng/ml vs. 252.716 ± 29.765 ng/ml, respectively) (Fig. 6A,B).

### Effect of controllable expression on reproductive performance of F0 transgenic pigs

Eight sows were impregnated by artificial insemination with semen collected from the founder transgenic pig and 83 F1 pigs were born. The average number of live pig births was approximately equal to the number of wild type live pig births during the same period (10.33 and 10.47, respectively). The 83 pigs included 42 transgenic pigs (26 male, 16 female) and 41 wild type pigs (22 male, 19 female); genotypes were detected molecularly.

### Production performance of F1 transgenic pigs

**Figure 7:**
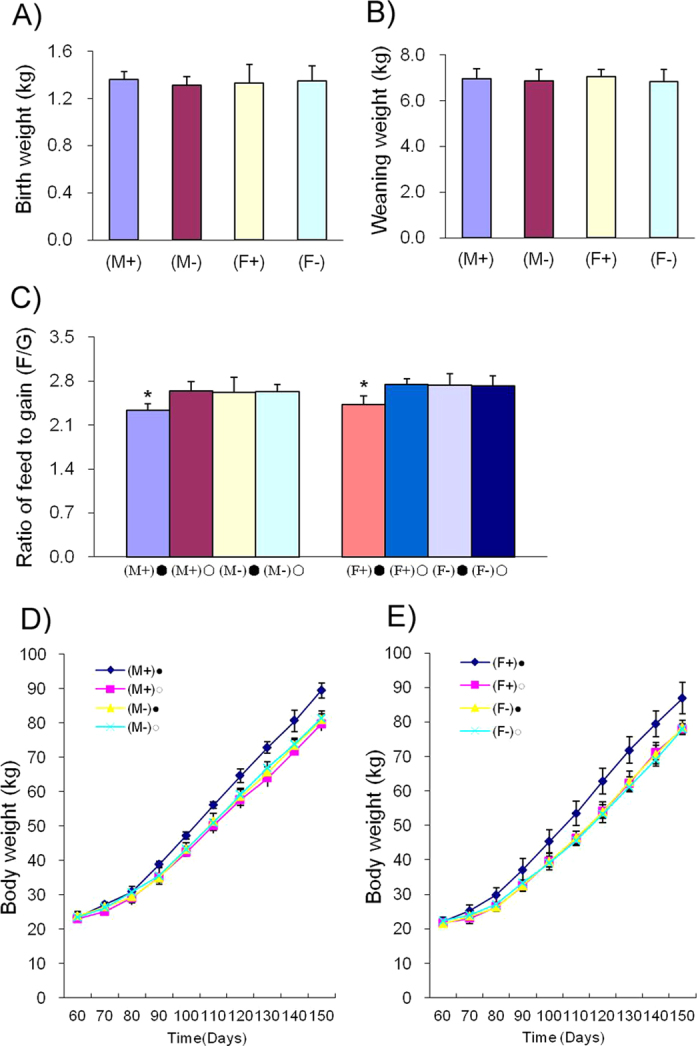
Growth rates and feed efficiencies of F1 transgenic and wild type pigs. **A**) Birth weights of F1 transgenic and wild type pigs. **B**) Weaning weights on day 28 of F1 transgenic and wild type pigs. **C**) Ratio of feed to gain. **D**) Growth curve of F1 transgenic male pigs. **E**) Growth curve of F1 transgenic female pigs. (M+)•: transgenic male F1 + DOX, (M+) •: transgenic male F1-DOX, (M-) •: wild type male F1 + DOX and (M-) •: wild type male F1-DOX; (F +) •: transgenic female F1 +DOX, (F +) •: transgenic female F1-DOX, (F -) •: wild type female F1 +DOX and (F -) •: wild type female F1-DOX.

**Figure 8:**
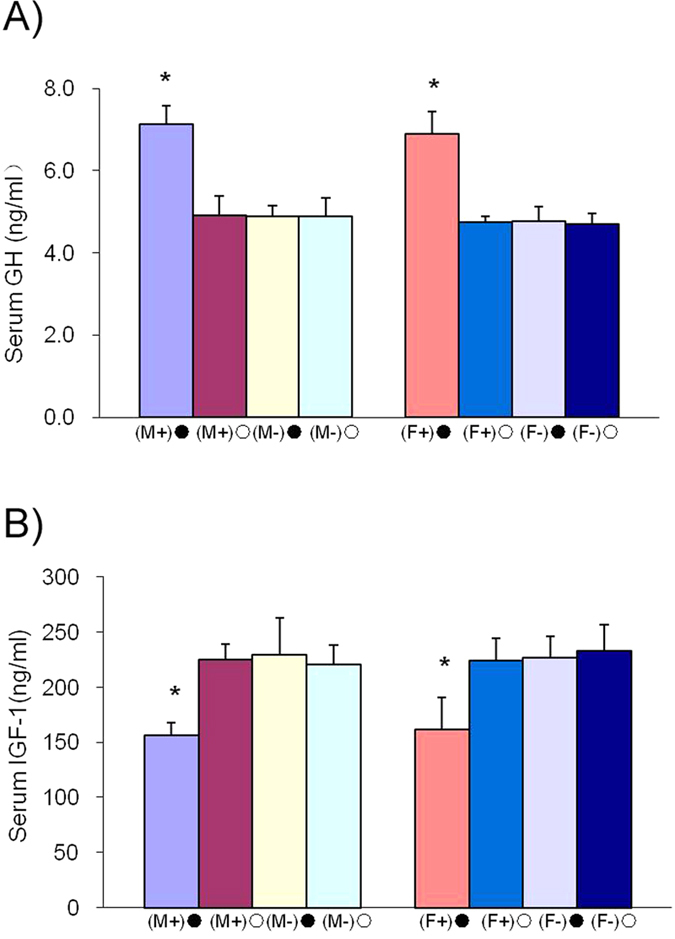
Serum pGH and IGF-1 levels in F1 transgenic pigs. **A**) Serum GH detection. The average serum pGH concentrations in the transgenic induction group (male and female) were significantly higher than those in the transgenic non-induction group and the wild type induction and non-induction groups. **B**) Serum IGF-1 detection. The average serum IGF-1 concentration in the transgenic induction group (male and female) decreased significantly compared to that in the transgenic non-induction group and the wild type induction and non-induction groups. There was no significant difference in serum IGF-1 levels in the transgenic non-induction group or the wild type induction and non-induction groups.

There was no significant difference between the birth weights of F1 generation transgenic pigs and F1 wild type pigs (males, 1.36 ± 0.03 kg vs.1.31 ± 0.03 kg; females, 1.33 ± 0.04 kg vs.1.35 ± 0.07 kg, respectively; P > 0.05). Similarly, weaning weights on day 28 were not significantly different between F1 transgenic and wild type pigs (males, 6.95 ± 0.17 kg vs. 6.87 ± 0.30; females, 7.06 ± 0.14 kg vs. 6.83 ± 0.17 kg, respectively; P > 0.05) (Fig. 7A,B). Pigs that were similar in weight at 65 days old were chosen for induction. Three months after beginning DOX induction, the ratio of feed to gain (F/G) was significantly lower in induced transgenic pigs (both males and females) than in non-induced transgenic pigs (males, 2.31 ± 0.06 vs. 2.64 ± 0.16 (F/G), respectively; P = 0.027 and females, 2.42 ± 0.14 (F/G) vs. 2.74 ± 0.10 (F/G), respectively; P = 0.028). There were no significant differences among transgenic or wild type pigs that did not undergo DOX induction or in wild type pigs that did undergo DOX induction (Fig. 7C). The body weights of F1 transgenic and wild type pigs were recorded and are shown in Fig. 7D,7E. The growth rate of DOX-induced, transgenic pigs were significantly higher, after DOX induction, than the growth rate of non-induced transgenic pigs. There were no obvious differences among the other three groups. During DOX induction, pGH levels increased significantly in the induction transgenic group compared with the non-induction transgenic, the wild type induction, and the wild type non-induction groups (males, 7.12 ± 0.45 ng/ml vs. 4.90 ± 0.47 ng/ml, 4.89 ± 0.26 ng/ml and 4.88 ± 0.46 ng/ml, respectively; P < 0.05 and females, 6.89 ± 0.56 ng/ml vs. 4.73 ± 0.16 ng/ml, 4.76 ± 0.35 ng/ml and 4.71 ± 0.26 ng/ml, respectively; P < 0.05) (Fig. 8A). Serum IGF-1 values declined significantly, during DOX induction, in the transgenic induction group compared with the transgenic non-induction, the wild type induction, and wild type non-induction groups (males, 156.66 ± 11.32 ng/ml vs. 224.91 ± 14.07 ng/ml, 229.61 ± 33.32 ng/ml and 220.72 ± 17.95 ng/ml, respectively; P < 0.05 and females, 161.61 ± 28.9 ng/ml vs. 224.28 ± 19.97 ng/ml, 227.72 ± 18.76 ng/ml and 233.34 ± 23.10 ng/ml, respectively; P < 0.05) (Fig. 8B).

### Slaughter performance of F1 transgenic pigs

**Figure 9:**
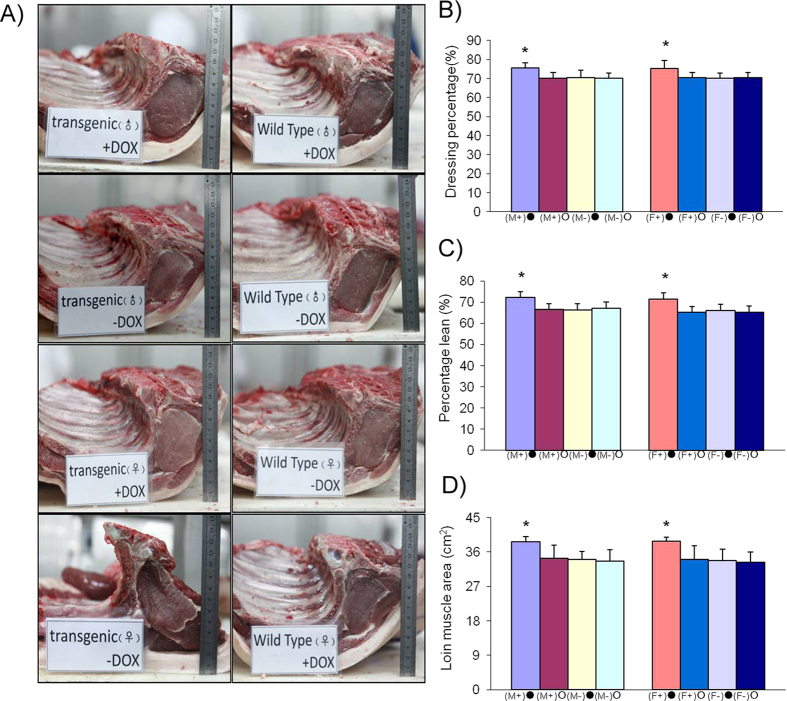
Slaughter measurements of F1 transgenic and wild type pigs. **A**) Cross sections through the loin at the 6^th^-7^th^ rib of F1 transgenic and non-transgenic pigs. **B**) Lean percentage in F1 transgenic and non-transgenic pigs. **C**) Dressing percentage (%) of F1 pigs. **D**) Loin muscle area (cm^2^) of F1 pigs. (F+)• and (F-)•, female F1 transgenic and wild type pigs induced with DOX; (F+) mn • and (F-) •, female F1 transgenic and wild type pigs without DOX; (M+) • and (M-) •, male F1 transgenic and wild type pigs induced with DOX; (M+) • and (M-) •, male F1 transgenic and wild type pigs without DOX.

At the conclusion of the induction experiment, 32 pigs were selected for a slaughter experiment (n = 8 in each group; 4 males and 4 females). The dressing percentages, carcass lengths, loin muscle areas and lean meat percentages were significantly higher (*P* < 0.05) in the transgenic induction group compared with the transgenic non-induction group and the wild type group. The average back fat in the transgenic induction group was lower than in the transgenic non-induction group and the wild type group, though not significantly. Skin depth was not significantly different among the 4 groups (*P* > 0.05) (Tables 1 and 2; Fig. 9).

### Meat quality of F1 transgenic pigs

Color, pH, tenderness and moisture were not significantly different among the 4 groups (*P* > 0.05) (Tables 3 and 4).

## Discussion

The traditional Tet-On regulatory system combines the rtTA and TRE systems, respectively, for transactivator expression and transactivator dependent expression of the target gene^44^. In transgenic mice, the rtTA and TRE systems are usually integrated in 2 different lines. When breeding, offspring are obtained in which the target gene can be regulated by the addition of DOX^45^. The length of time necessary for this approach makes it impractical for use in other mammalian species, including livestock^46^. The current study improves the operational efficiency of the traditional Tet-On system via integrating an rtTA cassette and a TRE cassette into a single vector and utilizing the HMC method to obtain transgenic cloned pigs with controllable pGH expression.

HMC is a nuclear transfer technique that is performed by hand. Compared with traditional SCNT, HMC is more efficient, needs fewer test conditions, requires a lower level of technical expertise and is very easy to use for large-scale production. Various breeds of transgenic cloned pigs have been successfully obtained using HMC^26^,^27^,^47^. In the current study, the success rate for obtaining blastocysts routinely reached 42–46% using the HMC method and the average number of cells per blastocyst was 83 on day 6. This level of efficiency is close to, or higher than, that obtained for other transgenic blastocysts prepared using the HMC method^27^,^48^,^49^, Furthermore, the developmental rate and cell number for blastocysts obtained using the HMC method in this study were far higher compared with traditional SCNT used in previous studies^50^,^51^ indicating that the foreign gene had no obvious deleterious effects on porcine embryonic development.

Many GH transgenic mice and pigs have been found to have higher growth rates and daily weight gains compared to controls. However, over-expression of the exogenous GH gene can induce a host of pathologic changes, including hepatomegaly, glomerular sclerosis, gastric ulcers, arthritis, infertility and premature death^43^,^52^,^53^,^54^,^55^. In this study, the induction test for the F0 generation was not done until day 65 in an effort to prevent the harmful effects on the health and reproductive performance of the animals, which can be caused by sustained premature expression of GH. In the current study, the expression levels of pGH were 34.6% higher after DOX induction than before induction. This modest increase may have resulted from the organism itself limiting excessive expression of pGH by foreign genes, so that growth was regulated according to age. In this study, a total of 5 F0 generation transgenic cloned pigs were obtained (3 males, 2 females), though the 2 sows died at 10–12 months of age as a result of disease. None of the 5 pigs demonstrated any abnormal symptoms after induction. The semen of the boars was collected for insemination of non-transgenic sows, which resulted in normal pregnancy rates and litter sizes. There was no onset of disease in F1 transgenic pigs prior to induction (the nursing period) or during induction (the fattening period). This result indicates that regulating the onset and level of GH expression can effectively reduce the harmful effects caused by sustained early expression.

Transgenic mice that express MTrGH, MThGH, or MTbGH genes grow at four times the rate of control mice during the maximum growth phase^41^,^56^. Yorkshire barrows were treated daily with pGH (22 μg/kg) for 30 consecutive days and their growth rates and feed efficiency improved by 10% and 4%, respectively^57^. Yorkshire-Duroc barrows (40 kg) that were treated daily with pGH (10, 30 and 70 μg/kg) for 35 consecutive days showed a significant increase in growth rate vs. controls (14% at 70 μg/kg) and a significant dose-dependent increase in feed efficiency (7% at 10 μg, 10% at 30 μg, 17% at 70 μg) vs. controls^58^. Compared to littermate controls, the growth rates and feed efficiencies of MTbGH transgenic pigs increased by 10–15% and 16–18%, respectively^42^. In this study, the growth rates in the DOX-induced transgenic group increased by 15.70% (males) and 11.49% (females), 15.87% (males) and 10.54% (females) and 14.53% (males) and 12.72% (females) compared with the non-induced transgenic and the wild type induced and non-induced males and females, respectively. These results are in accordance with those found with intramuscular injections of pGH and the foreign gene GH test (MTbGH), indicating that pGH from foreign genes can significantly enhance the growth rates and feed efficiencies of transgenic pigs through DOX-induced expression. In this study, the feed efficiencies of the DOX-induced transgenic pigs increased by 11.74% (males) and 11.63% (females), 11.06% (males) and 11.34% (females) and 11.4% (males) and 11.82% (females) compared with the non-induced transgenic and the wild type induced and non-induced males and females, respectively. The feed/gain ratio was lower than in the MTbGH transgenic pigs, but was consistent with the results from the intramuscular injection of pGH indicating that the inducible expression of foreign pGH has the same effect as that of an intramuscular injection of pGH. The meat quality of the pGH transgenic pigs was not significantly different than in pigs from the other groups. This suggests that the induced expression of a foreign pGH gene can increase the growth rate and feed efficiency of animals, while having no effect on meat quality.

In this study integrated controllable expression of a gene was obtained in a large transgenic, healthy population of pigs with normal reproductive capabilities. This could lead to improved feed efficiency, accelerated production processes and increased meat yields. This study lays the foundation for the development of new breeds of transgenic pigs and presents the successful application of controllable gene expression in transgenic livestock.

## Methods

### Chemical information

All chemicals and media were purchased from Sigma, Chemical Co. (St Louis, MO, USA). The primers used in this study were synthesized by Invitrogen Technology (Beijing, China). PCR reactions were performed using Taq DNA Polymerase (Takara, Cat#: DR001A, Dalian, China). Anti-Growth Hormone Polyclonal Antibody was purchased from Thermo Scientific Biotechnology (Cat#: PA1-85521, Waltham, MA USA). Polyclonal goat Anti-mouse IgG/HRPwas purchased from Santa Cruz Biotechnology (Cat#: 330, CA USA). Rabbit polyclonal antibody for beta Actin Loading Control (Cat#: PM053-7) was purchased from MBL. Dulbecco’s Modified Eagle’s medium (DMEM, Cat#: 12800-017) AND G418 (Cat#: 11811031) was purchased from Invitrogen (Grand Island, NY USA). The serum was purchased from Hyclone (Cat#: SV30087.02, Logan, UT, USA). Recombinant human basic fibroblast growth factor (bFGF) was purchased from Sigma (Cat#: F0291). Endo Free plasmid mini-prep kits were purchased from Omega Bio-Tek (Cat#: D6950, Norcross, GA USA). The restriction enzymes were purchased from Fermentas (Ontario, Canada).

### Animal studies

All animal experiments and procedures in this study were performed in accordance with the criteria outlined in the “Guide for the Care and Use of Laboratory Animals, Institute of Animal Sciences, Chinese Academy of Agricultural Sciences.” The procedures were approved by the Animal Care and Use Committee of the Germplasm Resource Center of Chinese Experimental Minipig (Permit Number: ACGRCM2008). All animals were housed in individual pens under controlled conditions (temperature, 18 °C–22 °C; relative air humidity, 30%–70%) and were fed twice daily on a restricted schedule and dietary dose [3% of body weight monthly; facility certification No.: SYXK (Beijing) 2008].

### Cell culture and vector fragment preparation

**Figure 10:**
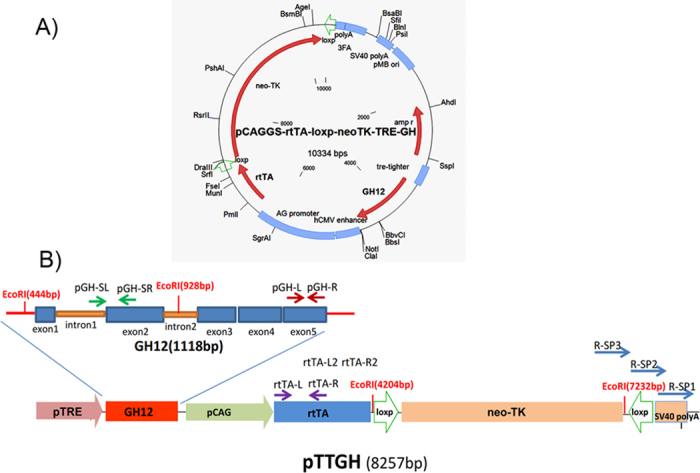
Schematic representation of the pCAGGS-rtTA-TRE-GH12 (pTTGH) vector fragment. **A**) Schematic of the pTTGH vector. **B**) pCAGGS-rtTA-loxp-neoTK-TRE-GH12 fragment (pTTGH, *Ssp* I, *Sfi* I cut, 8257 bp). The pTTGH vector was cut with restriction enzymes, *Ssp* I and *Sfi* I, and the pTTGH fragment (8257 bp) was recovered. The pTTGH fragment was constructed with the GH expression box, a GH12 gene controlled with a pTRE promoter, a GH expression inducing box and an rtTA gene expressed with a pCAG promoter. A fusion gene of a neomycin resistant gene and a diphtherial toxin (DTA) gene, flanked by loxP sites, was chosen as a selectable gene. The relative positions of the primer pairs pGH-SL/ pGH-SR, pGH-L/ pGH-R, rtTA-L/rtTA-R, rtTA-L2/rtTA-R2 and 3 sequence specific primers for genome walking are shown. The *EcoR*I restriction enzyme sites (444 bp, 928 bp, 4204 bp and 7232 bp) are shown above.

Porcine embryo fibroblast (PEF) cells were established from Large Yorkshire pigs. Fibroblasts were cultured in DMEM, supplemented with 15% FCS, 293 μg/ml L-glutamine, 100 U/ml penicillin, 100 μg/ml streptomycin and 5 ng/ml bFGF. The pTTGH vector (Fig. 10A) was cut with restriction enzymes, S*sp* I and S*fi* I, and the pTTGH DNA fragment (Fig. 10B) was recovered. The pTTGH DNA fragment was mixed with Lipo-fectamine2000 (Invitrogen, Cat#: 11668-019) and transfected into PEF cells, according to the manufacturer’s protocol. The pTTGH vector was cut with S*sp* I and S*fi* I, and the pTTGH fragment (8257 bp) was recovered. The pTTGH fragment was constructed with the GH expression box and the GH12 gene was controlled with the pTRE promoter and the GH expression-inducing box. The rtTA gene was expressed with the pCAG promoter. The fusion gene of a neomycin resistant gene and a diphtherial toxin (DTA) gene, flanked by the lox P sites, was chosen as the selectable gene. The relative positions of the pairs pGH-SL/pGH-SR, pGH-L/pGH-R and rtTA-L/rtTA-R and of the 3 sequence-specific primers used for genomic walking are shown in Fig. 1B. The E*co*R I restriction enzyme sites are shown in Fig. 1B.

### Generation of transgenic donor cells

Cells were separated from 35 day-old Large White fetuses. The PEF cell lines were established and the SRY gene was detected with PCR for identification of sex. Porcine ear skin fibroblast (pESF) cells, from both boars and sows, were chosen as controls. The primers used were as follows, SryF (5′ - CCGACGGACAATCATAGC - 3′ , forward) and SryR (5′ - GGTGGATGTTACCCTACTGT - 3′ , reverse). G418-selected PEF cells were cultured in DMEM with 10% FBS. pGH mRNA was measured before and after DOX induction (1000 ng/μl DOX concentration in the culture medium). Partial cells were analyzed by PCR and RT-PCR for verification of transgene construction. Genomic DNA from the cells was amplified for rtTA using the following specific primer pairs rtTA-L (5′ - CATTCCGCTGTGCTCTCCTCTC - 3′ , forward) and rtTA-R(5′ - GAGCGTCAGCAGGCAGCATATC - 3′ , reverse) under the following PCR conditions: 30 cycles of 95 °C for 30 sec, 57 °C for 35 sec and 72 °C for 40 sec. For the detection of GH and rtTA transcripts, total cellular RNA was isolated from each candidate clone using TRIzol reagent (Invitrogen) and the cDNA was synthesized using the Revert Aid First Strand cDNA Synthesis Kit (MBI Fermentas), according to the manufacturer’s protocol. The 4 cell clones with the highest rtTA expression levels were chosen for the detection of GH gene expression before and after DOX-induction. RT-PCR was performed using the following primer pairs, rtTA-L (5′ - TACACTGGGCTGCGTATTGGAG - 3′ , forward) and rtTA-R (5′ – ATCGGCTGGGAGCATGTCTAAG - 3 ′ , reverse); pGH detection primers and housekeeping gene GAPDH, pGH-L (5′ - GGGCAGGACAGATCCTCAAG - 3′ , forward) and pGH-R (5′ - GACCCGCAGGTATGTCTCAG - 3′ , reverse); pGAPDH-L (5′ - AGCAATGCCTCCTGTACCAC - 3′, forward) and pGAPDH-R (5′ - AAGCAGGGATG ATGTTCTGG - 3′ , reverse). The QRT-PCR kit (Takara, Cat#: DRR081A) reaction system and reaction temperature were used. The 2 cell clones with the highest induced efficiencies were cultivated for Western blot assays. Western blots of GH protein in porcine primary fibroblasts were done using an anti-growth hormone polyclonal antibody. Western blots were performed, as described previously^27^,^59^.

### Chromosome walking and QRT-PCR analysis of the transgene in cells

The GH copy number in transgenic cells was detected with QRT-PCR. The 2 endogenous copies of pGH were chosen as a reference. Cellular DNA was chosen as a template for QRT-PCR. QRT-PCR was performed using the following primer pairs: pGH detection primers and the housekeeping gene transferrin receptor gene (TFRC), pGH-L (5′ - GGGCAGGACAGATCCTCAAG - 3′ , forward) and pGH-R (5′ - GACCCGCAGGTATGTCTCAG - 3′ , reverse); pTFRC-L (5′ - GAGACAGAAACTTTCGAAGC - 3′ , forward) and pTFRC-R (5′ - GAAGTCTGTGGTATCCAATCC - 3′ , reverse). The foreign pTTGH insertion sites and their chromosomal positions in the transgenic cell lines were detected using the genome walking method. Three sequence-specific primers were designed based on the right side of the pTTGH sequence as follows, R-SP1 (5′ - CCGGATACCTGTCCGCCTTTCTC - 3′) , R-SP2 (5′ - GTGGCGCTTTCTCATAGCTCACG - 3′) and R-SP3 (5′ - TGCGCCTTATCCCGGTAACTATCG - 3′). Overlapping PCR was performed according to the Genome Walker kit protocol (Takara, Cat#: D316). Specific amplified products were recovered via gel extraction (BioTeke, Cat#: DP4101, Beijing, China), linked with the vector pGEM-T (Promega, Cat#: A1360, Madison, USA) and then transformed into competent cell *E*. *coli.* (Transgen, Cat#: CD201-01). The insertion sequence was sequenced and compared with the whole pig genome sequence in GenBank.

### Production of embryos obtained by HMC

PEF cells were grown in DMEM supplemented with 10% fetal bovine serum (FBS; HyClone) (batch number NRE0007) at 38.5 °C and 5% CO_2_. Somatic cell preparation was performed as described previously^27^,^60^. The chromosomal count of the established fibroblast cell line indicated that the donor cells were of a normal diploid karyotype. The preparation of recipient oocytes (maturation, cumulus/zona removal and manual enucleation) and the fusion, activation and culture of NT embryos were done, as previously described^27^,^60^.

### PCR and Southern blot analysis of the transgene in F0 pigs

F0 pigs were validated by PCR and Southern blot analysis using genomic DNA extracted from the tail and screened as described above. The primers and PCR cycling conditions for GH and rtTA are described above. Genomic DNA (40 μg) from F0 pigs was digested overnight with E*co*RI (MBI Fermentas), separated through a 1% agarose gel and alkali-transferred onto Hybond-N+ nylon membranes (Amersham Pharmacia Biotech, Buckinghamshire, UK), as described previously^61^. Probes were PCR-amplified from pTTGH DNA fragments using validated GH and rtTA primers, pGH-SL (5′ - CACCAACCTTGGGCTTTGGG - 3′ , forward) and pGH-SR (5′ - CACGGCGTTGGCAAATAGGC - 3′ , reverse), rtTA-L (5′ - CTGTGCTCTCCTCTCACATC- 3′ , forward) and rtTA-R (5′ - GAATCGGTGGTAGGT GTCTC - 3′ , reverse), which were digoxigenin (Dig)-labeled using a nick translation system (Innogent-cn, Shenzhen, China), according to the manufacturer’s instructions. The Dig-labeled DNA was detected via chemiluminescence using streptavidin alkaline phosphatase and CDP-Star® (Innogent-cn). Hybridization, membrane washing and probe detection were done according to the manufacturer’s instructions.

### Transgene induction studies in F0 pigs

Ear-tip fibroblasts were separated from transgenic pigs and grown in DMEM supplemented with 10% fetal bovine serum at 38.5 °C and 5% CO_2_. Total RNA was extracted from the cells prior to and 36 h after the addition of 1000 ng/μl DOX to culture medium. Total protein was extracted from the cells prior to and 48 h after DOX induction. GH mRNA expression was measured before and after induction with 1000 ng/μl DOX in DMEM. QRT-PCR was performed using pGH primer pairs and conditions as described in the manufacturer’s protocol. Western blots of GH protein in porcine primary fibroblasts were done as previously described. On day 150, 3 founder male pigs were induced by intramuscular injection of DOX and 3 non-transgenic male Large Yorkshire pigs were selected as a control group. DOX (20 mg/kg body weight) was injected intramuscularly, once daily for two weeks. Blood samples (3 to 4 ml each) were collected from each pig 3 times per day, before feeding (at 6:00, 12:00 and 18:00), on 3 separate days before and after DOX injection. Separate serum samples were used to measure IGF-1 levels (HY-082, Sino-UK institute of Biological Technology Beijing, China) and GH levels (HY-037, Sino-UK institute of Biological Technology, Beijing, China) via the radioimmunoassay (RIA) method, according to the manufacturer’s protocol^61^.

### Induction of F1 transgenic pigs

Semen was artificially collected from founder transgenic pigs and used to artificially inseminate non-transgenic sows in estrus. F1 generation pigs (including transgenic and non-transgenic pigs) were detected with PCR, Southern blot and QRT-PCR. The number of live pig births, as well as the birth and weaning weights, were recorded. Transgenic and non-transgenic F1 generation pigs, with similar body weights, were chosen for the DOX induction test. The pigs were divided into 4 groups: DOX-induced transgenic pigs (13 males, 7 females), non-induced transgenic pigs (10 males, 6 females), DOX-induced, non-transgenic pigs (8 males, 8 females) and non-induced, non-transgenic pigs (8 males, 8 females). DOX (Hua Nan Animal health products co., LTD, Shang Hai) was added to the feed (5 mg/kg body weight, per day) beginning when animals were 65 days of age. The weight of each pig was recorded every 10 days and was taken prior to feeding. Blood samples were also collected every 10 days. Serum was obtained by centrifugation and stored at −80 °C until analysis for GH and IGF-1 levels.

### Slaughter experiment and meat quality analysis of F1 transgenic pigs

The F1 pigs, including transgenic and non-transgenic pigs, were divided into 4 groups, as described above, and 32 pigs (n = 8 for each group, 4 males and 4 females) were slaughtered. Prior to the first day of the slaughter test, blood samples were collected via the anterior vena cava at 6:00, 12:00 and 18:00. Samples were pooled for each pig and were assayed for GH and IGF-1. The carcass measurements were obtained from the left side and included loin muscle area, first-rib back fat thickness, 10^th^-rib back fat thickness, last-rib back fat thickness, last-lumbar vertebra back fat thickness, carcass length and muscle score. Average back fat was determined by averaging the back fat thickness at the first rib, between the 6^th^ and 7^th^ ribs, at last rib and at the last lumbar vertebrae. Loin muscle area was determined by tracing the loin muscle surface area at the 10^th^ rib and the area was determined with a compensating polar planimeter. Approximately 45 min and 24 h after slaughter, pH measurements were taken on the right side of the carcass, in the loin muscle, and between the 10^th^ and 11^th^ ribs. The pH of the loin muscle was determined using a hand-held pH meter fitted with a spear-tipped electrode. The meat quality scores (color and firmness-wetness) were also determined on the 10^th^ rib chop. Water-holding capacity was determined, in triplicate, from samples obtained at the 9^th^ rib chop, using a press method.

### Statistical analysis

Statistical analysis was performed using the computerized package generated by SPSS 16.0 software for Windows. All values are presented as the mean± S.E.M. The statistical significance between groups was analyzed by one-way ANOVA and *P* values < 0.05 were considered significant. All experiments were repeated at least three times.

## Additional Information

**How to cite this article**: Ju, H. *et al*. The transgenic cloned pig population with integrated and controllable GH expression that has higher feed efficiency and meat production. *Sci. Rep.* **5**, 10152; doi: 10.1038/srep10152 (2015).

## Supplementary Material

Supplementary Information

## Figures and Tables

**Table 1:** Carcass traits of F1 transgenic and wild type pigs (male).

**Male**		**Dressing percentage (%)**	**Carcass length (cm)**	**Average back fat (cm)**	**Skin depth (cm)**	**Loin muscle area (cm2)**	**Percentage lean (%)**
transgenic	+Dox	75.5 ± 2.65^a^	93.5 ± 4.40^a^	1.97 ± 0.31	0.20 ± 0.02	38.73 ± 1.29^a^	72.3 ± 2.63^a^
	−Dox	70.3 ± 2.83^b^	86.0 ± 2.94^b^	2.20 ± 0.24	0.23 ± 0.03	34.40 ± 3.44^b^	66.4 ± 2.78^b^
wild type	+Dox	70.5 ± 3.90^b^	87.0 ± 3.37^b^	2.20 ± 0.24	0.24 ± 0.02	34.10 ± 2.05^b^	66.2 ± 2.95^b^
	−Dox	70.1 ± 2.90^b^	87.0 ± 2.58^b^	2.15 ± 0.25	0.23 ± 0.02	33.65 ± 2.99^b^	67.2 ± 2.89^b^

Note: Means in a column with different superscripts are significantly different (P < 0.05).

**Table 2:** Carcass traits of F1 transgenic and wild type pigs (female).

**Female**		**Dressing percentage (%)**	**Carcass length (cm)**	**Average back fat (cm)**	**Skin depth (cm)**	**Loin muscle area (cm2)**	**Percentage lean (%)**
transgenic	+Dox	75.3 ± 4.06^a^	86.5 ± 2.65^a^	2.01 ± 0.11	0.21 ± ± 0.03	33.78 ± 1.02^a^	71.43 ± 2.98a
	−Dox	70.4 ± 2.89^b^	80.0 ± 3.65^b^	2.20 ± 0.24	0.23 ± 0.03	34.10 ± 3.58^b^	65.05 ± 2.89^b^
wild type	+Dox	70.3 ± 2.50^b^	78.0 ± 3.74^b^	2.25 ± 0.24	0.21 ± 0.03	33.68 ± 3.04^b^	66.05 ± 2.80^b^
	−Dox	70.4 ± 2.73^b^	80.5 ± 3.70^b^	2.25 ± 0.24	0.24 ± 0.01	33.25 ± 2.75^b^	65.25 ± 2.89^b^

Note: Means in a column with different superscripts are significantly different (P < 0.05).

**Table 3:** Meat quality traits of F1 transgenic and wild type pigs (male).

**Female**		**pH**	**Color**			**Tenderness (kgf)**	**Moisture (%)**
			**L**	**A**	**B**		
transgenic	+Dox	6.32 ± 0.03	50.84 ± 2.45	11.75 ± 1.24	8.36 ± 1.32	8.16 ± 0.95	76.69 ± 3.23
	−Dox	6.42 ± 0.05	49.19 ± 3.23	11.90 ± 2.10	8.80 ± 1.34	8.22 ± 1.23	76.36 ± 2.36
wild type	+Dox	6.23 ± 0.07	47.26 ± 3.64	12.97 ± 1.23	7.14 ± 1.23	7.81 ± 1.12	77.00 ± 1.87
	−Dox	6.34 ± 0.04	49.50 ± 3.21	10.79 ± 1.74	8.83 ± 1.12	7.85 ± 0.94	75.76 ± 2.31

**Table 4:** Meat quality traits of F1 transgenic and wild type pigs (female).

**Female**		**pH**	**Color**			**Tenderness (kgf)**	**Moisture (%)**
			**L**	**A**	**B**		
transgenic	+Dox	6.25 ± 0.03	41.92 ± 1.23	12.76 ± 0.89	6.35 ± 0.23	5.56 ± 0.23	76.86 ± 2.56
	−Dox	6.38 ± 0.02	53.92 ± 1.32	11.24 ± 0.74	7.33 ± 0.34	3.92 ± 0.32	77.52 ± 1.87
wild type	+Dox	6.32 ± 0.06	52.90 ± 1.53	10.69 ± 0.67	8.44 ± 0.45	3.52 ± 0.36	74.84 ± 2.16
	−Dox	6.41 ± 0.07	50.60 ± 1.25	10.91 ± 0.12	8.47 ± 0.35	5.91±0.54	75.41 ± 1.95
