# Supplementary material for: The transgenic cloned pig population with integrated and controllable GH expression that has higher feed efficiency and meat production
Source: Sci Rep. 2015 May 11;5:10152. doi: 10.1038/srep10152 (PMC5386205; doi:10.1038/srep10152)
Supplement: Supplementary Information [file srep10152-s1.doc]

**Title: The transgenic cloned pig population with integrated and controllable GH expression that has higher feed efficiency and meat production**

Huiming Ju, Jiaqing Zhang, Lijing Bai, Yulian Mu, Yutao Du, Wenxian Yang, Yong Li, Anzhi Sheng, Kui Li

Supplementary Figure and figure legends


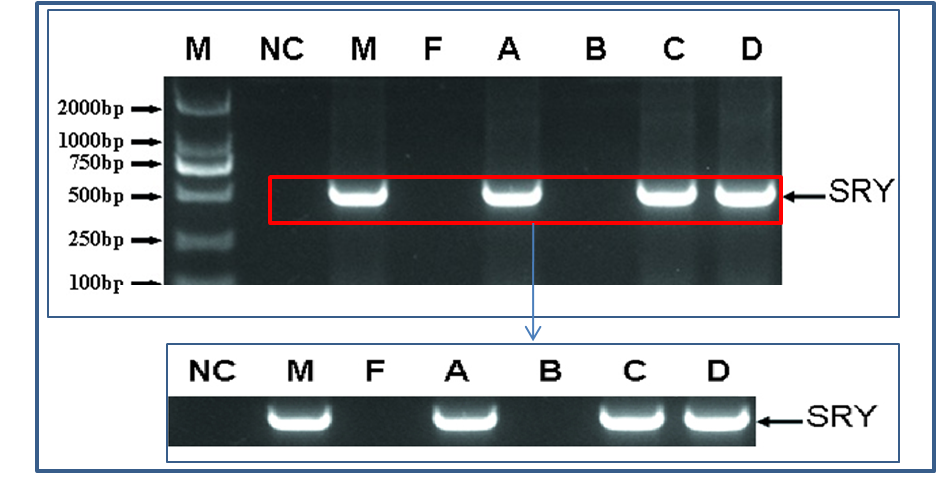
Supplementary Figure S1. Full-length gels for Figure2. B).


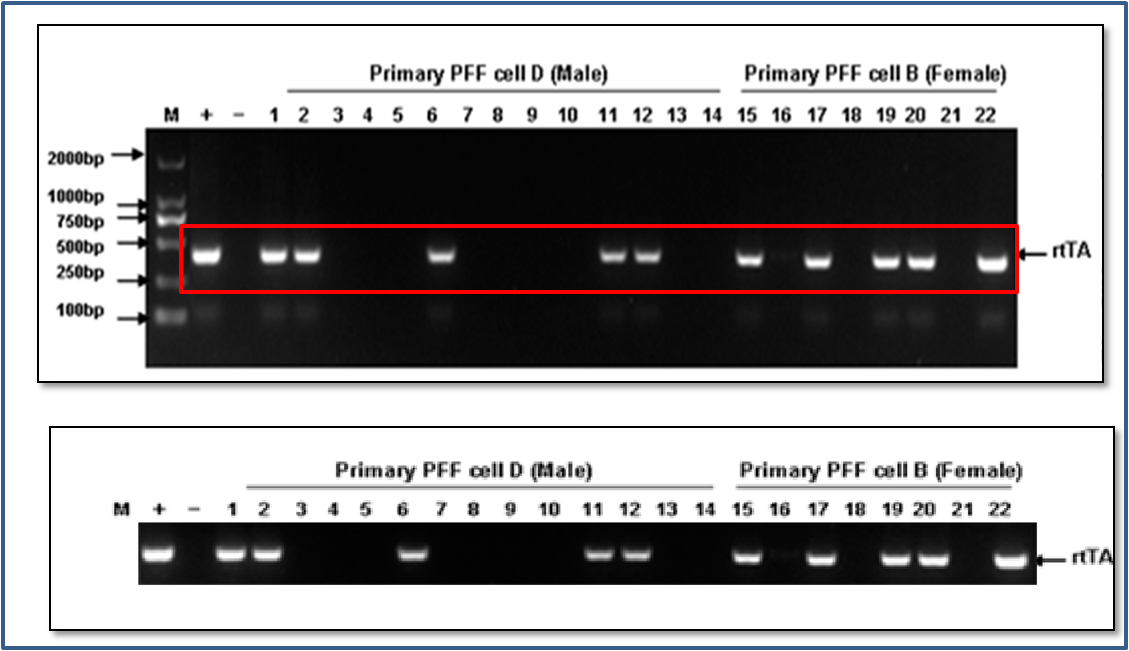
Supplementary Figure S2. Full-length gels for Figure 3. A)


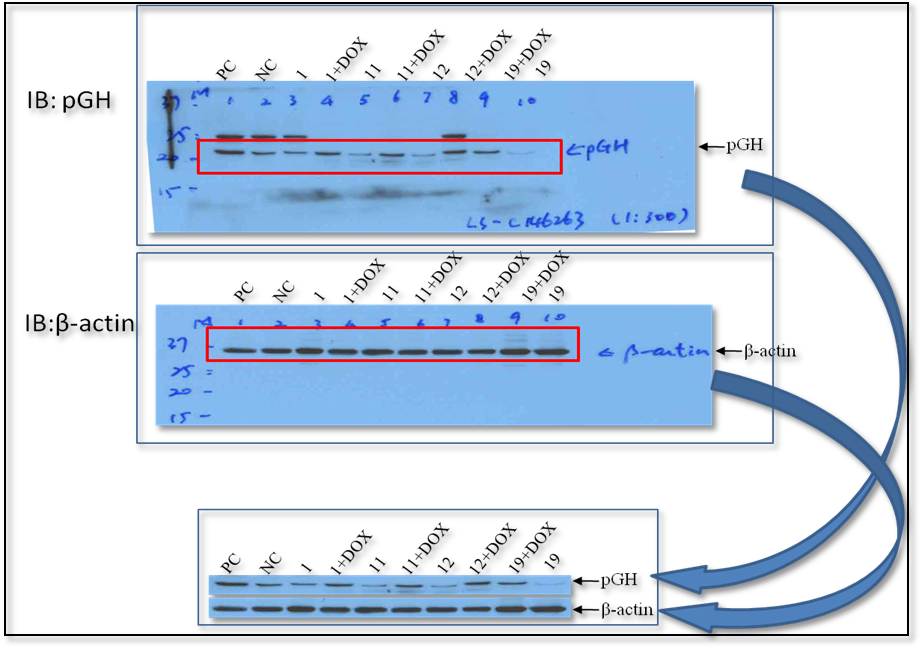
Supplementary Figure S3. Full-length gels for Figure3. D).

Supplementary Figure S4. Full-length gels for Figure 5. A)
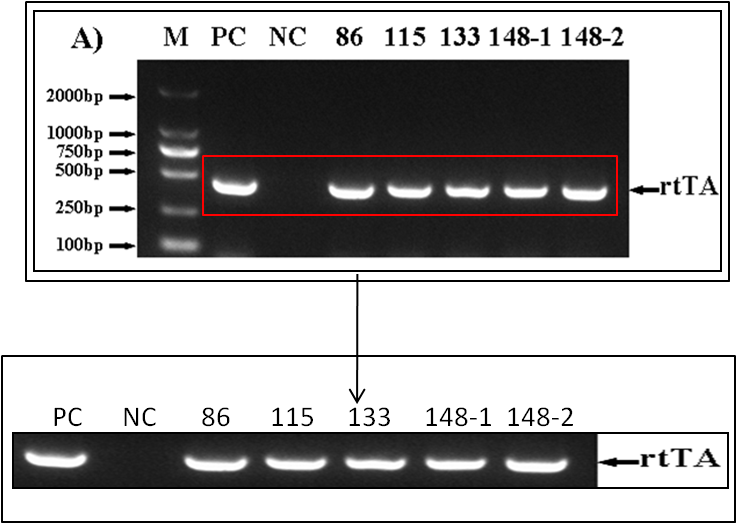
.

Supplementary Figure S5. Full-length gels for Figure 5. B).


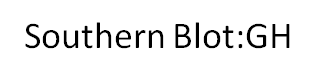

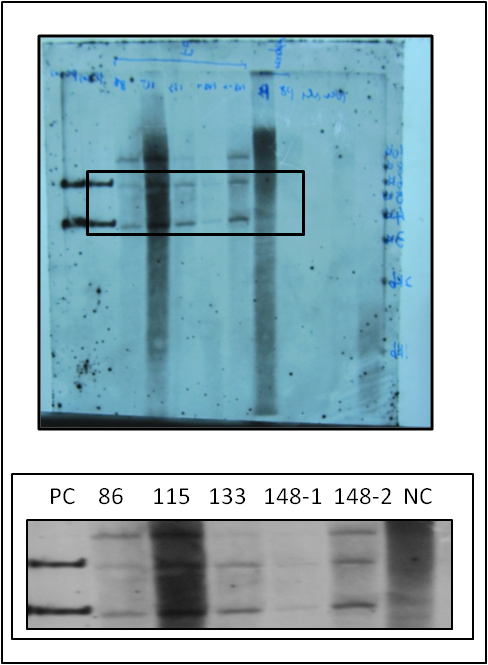


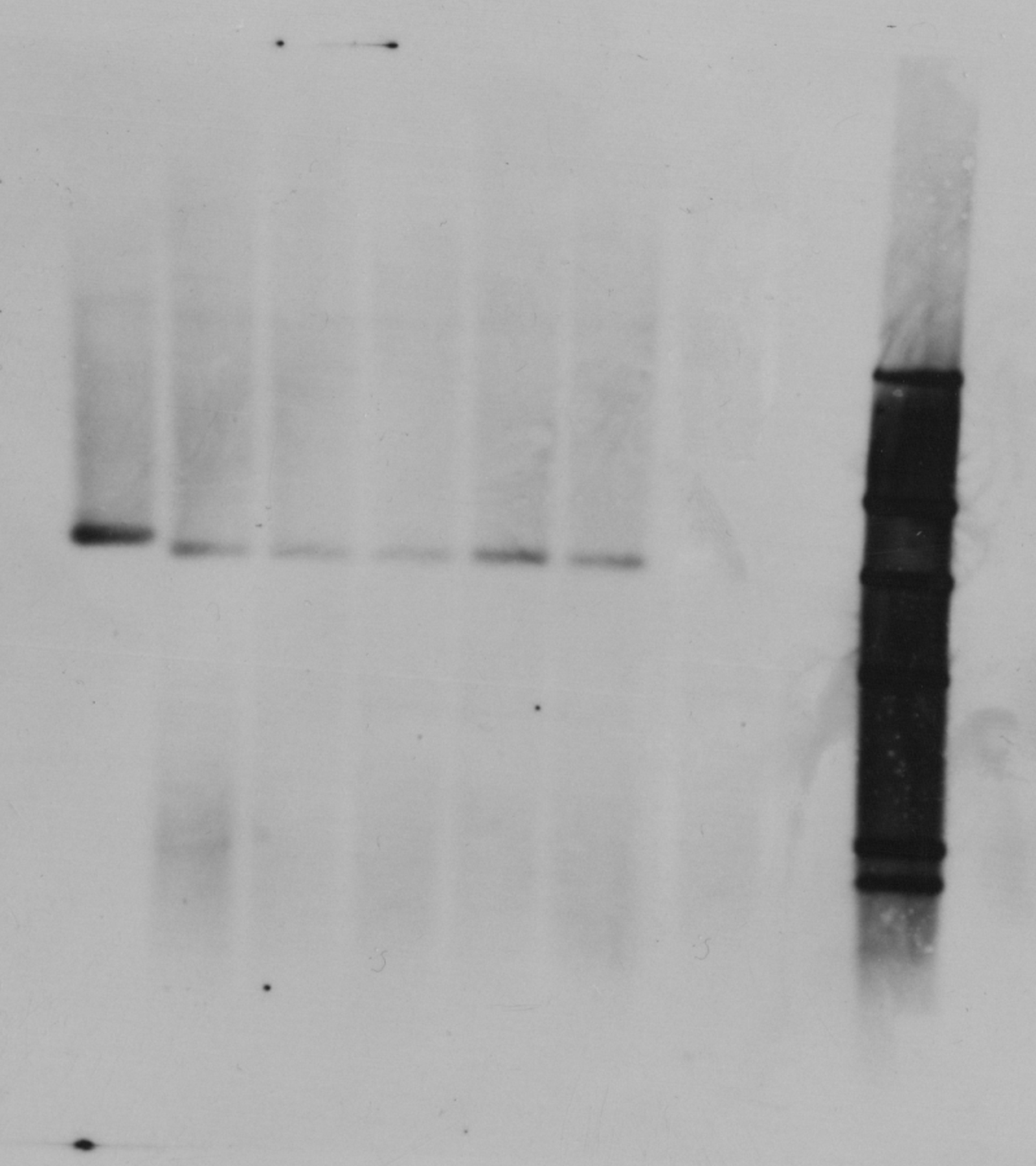

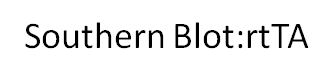


PC 86 115 133 148-1 148-2 NC

PC 86 115 133 148-1 148-2 NC


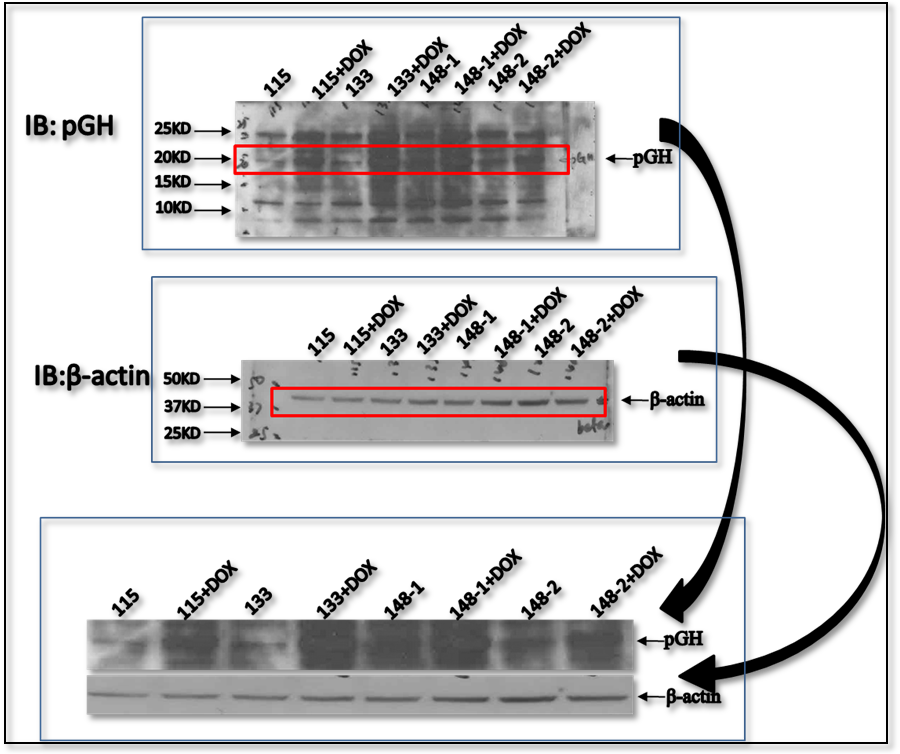
Supplementary Figure S6. Full-length gels for Figure 6. C).
